# Characterization of *Pythium* Transcriptome and Gene Expression Analysis at Different Stages of Fermentation

**DOI:** 10.1371/journal.pone.0065552

**Published:** 2013-06-18

**Authors:** Yuanmin Zhu, Pengpeng Zhou, Jingrong Hu, Ruijiao Zhang, Liang Ren, Maoteng Li, Fan Ning, Wei Chen, Longjiang Yu

**Affiliations:** 1 Institute of Resource Biology and Biotechnology, Department of Biotechnology, College of Life Science and Technology, Huazhong University of Science and Technology, Wuhan, China; 2 Key Laboratory of Molecular Biophysics Ministry of Education, Huazhong University of Science and Technology, Wuhan, China; 3 Wuhan Institute of Biotechnology, Wuhan, China; 4 Department of Environmental and Bio-chemical Engineering, Wuhan Vocational College of Software and Engineering, Wuhan, China; Auburn University, United States of America

## Abstract

**Background:**

The *Pythium splendens* is a potentially useful organism for the synthesis of large amounts of eicosapentaenoic acid. Peak biomass and lipid accumulation do not occur at the same time and growth temperature has an effect on the fatty acid composition. Little is known about the pathway or the genes involved in growth, lipid synthesis or temperature resistance in *P. splendens*. Analysis of the transcriptome and expression profile data for *P.splendens*RBB-5 were used to extend genetic information for this strain and to contribute to a comprehensive understanding of the molecular mechanisms involved in specific biological processes.

**Methodology/Principal Findings:**

This study used transcriptome assembly and gene expression analysis with short-read sequencing technology combined with a tag-based digital gene expression (DGE) system. Assembled sequences were annotated with gene descriptions, such as gene ontology (GO), clusters of orthologous group (COG) terms and KEGG orthology (KO) to generate 23,796 unigenes. In addition, we obtained a larger number of genes at different stages of fermentation (48, 100 and 148 h). The genes related to growth characteristics and lipid biosynthesis were analyzed in detail. Some genes associated with lipid and fatty acid biosynthesis were selected to confirm the digital gene expression (DGE) results by quantitative real-time PCR (qRT-PCR).

**Conclusion/Significance:**

The transcriptome improves our genetic understanding of *P.splendens*RBB-5 greatly and makes a large number of gene sequences available for further study. Notably, the transcriptome and DGE profiling data of *P.splendens*RBB-5 provide a comprehensive insight into gene expression profiles at different stages of fermentation and lay the foundation for the study of optimizing lipid content and growth speed at the molecular level.

## Introduction

Eicosapentaenoic acid (EPA; 5,8,11,14,17-*cis*-eicosapentaenoic acid) is an ω-3 C_20_-polyunsaturated fatty acid (PUFA) with a wide range of functions in biological systems. EPA and its metabolites are used as biomedical products and in nutraceuticals, fortified foods and health supplements [1], [2]. EPA is important in the treatment of cardiovascular disease [3]–[5] and in geriatrics, such as Alzheimer's disease [4] and the prevention of cognitive decline in the elderly [6]. EPA is effective also in arresting and minimizing tumor growth and metastasis, such as breast cancer [7]. Currently, there is a great demand for EPA worldwide.

Marine fish oil is the major source of EPA but the world's fish stocks are declining owing to overfishing and are failing to meet the increasing demand for purified EPA. Marine sources are at risk of containing methyl mercury and other chemical contaminants [8]–[10]. Thus, a sustainable source(s) of EPA is needed. The potential of a transgenic plant [11], [12] for EPA production was studied owing to the high oil content in the seed, and several genes (Δ5, Δ6 and ω-3 fatty acid desaturases etc.) were introduced into the plant, the yields of PUFAs are high but the growth period of the plant is lengthy and is constrained by geography. EPA produced by microorganisms is currently the main focus of study because of the simple culture conditions required, the brief culture cycle and the high yields of EPA. Some microorganisms, including microalgae [13], bacteria [14] and fungi [15], have the ability to synthesize EPA but there are some problems associated with their use, such as a lengthy fermentation stage and a low biomass, to overcome before mass production is feasible. *Mortierella*, for example, has a high yield of EPA but this is accompanied by an even greater yield of arachidonic acid (AA), which is difficult to separate from EPA. The production of EPA by the filamentous fungi *Pythium* spp. has attracted much attention. *Pythium* is specific for Oomycetes, which are found in both terrestrial and aquatic environments. *Pythium* spp. all produce EPA but yield varies among species; *P. ultimum* [16] and *P. irregular* [17] are among the most promising candidates. A study of the growth and biosynthesis of EPA by *P. irregular* [18] shows accumulation of ω-3 PUFAs is greater in cultures grown at low temperature (12∼14°C), which also was verified by *Mortierella* [19]. The strain *P. splendens*RBB-5, which we isolated from soil on the basis of polymerase chain reaction (PCR) amplification [20], has the ability to produce EPA and low temperature (15°C) promotes the synthesis of EPA; however, the yield of EPA is not high enough for industrial application. Some *Pythium* lipid synthesis genes are cloned and their functions are known (e.g. Δ5, Δ6 and Δ17 fatty acid desaturases) [21]–[23], but a comprehensive analysis of the mechanisms underlying growth and biosynthesis of lipids and EPA is needed to aid improvement of the EPA yield.

The next generation of high-throughput and cost-effective sequencing technology has emerged over the past few years [24]–[26]. RNA-Seq refers to whole transcriptome shotgun sequencing in which mRNA or cDNA is fragmented mechanically and the overlapping short fragments cover the entire transcriptome. RNA-Seq, which is not dependent on an earlier description of the genomic sequence of the target species, has improved the efficiency and speed of new gene discovery dramatically [27]–[31]. DGE involves tag-based transcriptome sequencing and the short raw tags are generated by an endonuclease [32]. The powerful combination of RNA-Seq and DGE combines the advantages of both. Large-scale functional assignment of genes is obtained by assembly of a large sequenced transcriptome library and quantitative gene expression comparisons are unbiased. A total of 26,308 unique sequences are annotated and 11,476 of these are assigned to specific metabolic pathways of *Siraitia grosvenorii* by RNA-Seq and the genes involved are identified by DGE analysis [33]. RNA-Seq and DGE are well suited for surveying the complexity of transcription and discovering new genes, for indicating a large set of novel environment response genes and for comparing gene expression profiles in different species [34].

In this study, 14,518,964 bases of high-quality DNA sequence were generated from equal amounts of RNA taken from *P. splendens*RBB-5 at 48 h, 100 h and 144 h with Illumina/Solexa technology. This shows the feasibility of short-read sequencing used for the de novo assembly and annotation of genes expressed in eukaryotes without prior genome information. In all, 23,796 unigenes of *P. splendens*RBB-5 are known. Construction of three DGE libraries (48 h (2-1), 100 h (4-1) and 148 h (6-1)) allows comparison of the gene expression profiles of *P. splendens*RBB-5 at different stages of fermentation. The assembled, annotated transcriptome sequences and gene expression profiles provide useful information about the pathway at different stages of fermentation and identification of genes involved in growth and biosynthesis of lipids. The results improve the overall view of the *P. splendens*RBB-5 transcriptome and offer the possibility of further analysis.

## Materials and Methods

### *P. splendens*RBB-5 collection and preparation

*P. splendens*RBB-5 was isolated from soil based on polymerase chain reaction amplification [20]. It is maintained on potato dextrose agar (PDA: glucose 30 g/L, potatoes extract 200 g/L and agar 20 g/L) slants at 4°C and is transferred every 2 months. The medium containing: glucose 80 g/L, yeast extract 5 g/L, NaNO_3_ 4 g/L, KH_2_PO_4_ 3 g/L, MgSO_4_ 0.5 g/L. The pH is adjusted to 5.0 before autoclaving at 121°C for 20 min. In preparation for an experiment, a stock culture is vigorously stirred in 50 mL sterilized water with glass beads for 30 min, and 6 milliliters of mycelia suspension transferred to 250 mL shake flasks (covered with 8 layers of cheese cloth) containing 80 mL medium. This is followed by incubation at 25°C for 96 h and 15°C for 52 h with shaking at 180 rpm. The Mycelia is collected by filtration and weighed. The samples were immediately frozen in liquid nitrogen and stored at −80°C until further processing.

### RNA isolation and library preparation for transcriptome analysis

Total RNA was isolated using the fungal RNA kit (Omega). The RNA samples were treated with DNase at a concentration of 1 unit/µg of total RNA and purified using RNA clean Kit (TIANGEN). To obtain complete gene expression information, a pooled RNA sample including different fermentation stages (48 h, 100 h and 148 h) was used for transcriptome analysis. The RNA integrity was confirmed using the 2100 bioanalyzer (Agilent technologies).The RNA beads with Oligo (dT) were used to isolate poly (A) mRNA after total RNA was collected from eukaryote. The samples were prepared using Illumina HiSeq™ 2000 following manufacturer's recommendations for transcriptome analysis. Briefly, the mRNA (a mixture of RNA from different fermentation stages at 48 h, 100 h and 148 h) was interrupted to short fragments by adding fragmentation buffer. Then by taking these short fragments as templates, random hexamer-primer was used to synthesize the first-strand cDNA. The second-strand cDNA was synthesized using buffer, dNTPs, RNaseH and DNA polymerase I respectively. The short fragments were purified with QiaQuick PCR extraction kit and resolved with EB buffer for end reparation and adding poly (A). After that, the short fragments were connected with sequencing adapters. The suitable fragments were selected for the PCR amplification as templates after the agarose gel electrophoresis. The transcriptome data of *P. splendens* were submitted to sequence read archive (SRA) (accession number SRR643895) at the NCBI.

### Analysis of Illumina sequencing results

The cDNA library was sequenced on the Illumina sequencing platform (GAII). The raw reads from the images were generated using Illumina GA pipeline 1.3. The raw reads that only had 3′adaptor fragments were removed and a coverage length of 100 bp was assembled using SOAPdenovo [35]. The generated unigenes larger than 200 bp were analyzed by searching the GenBank database with BLASTX algorithm (http://www.ncbi.nlm.nih.gov/). Gene ontology (GO) and KEGG ontology (KO) annotations of the unigenes were determined using Blast2GO [36], WEGO software [37] and EST scan software [38].

### DGE library preparation and sequencing

The total RNA was extracted from different fermentation stages (48 h, 100 h, 148 h) using the fungal RNA Kit (Omega). DEG libraries were prepared using the Illumina gene expression sample prep kit and Illumina sequencing chip (flowcell). Briefly, poly (A)^+^ RNA was purified from 6 mg of total RNA using oligo (dT) magnetic beads. Then double-stranded cDNA were transferred through reverse transcription using 4 base recognition enzymes NlaIII to digest this cDNA, and Illumina adaptor 1 was ligated. Mmel was used to digest at 17 bp downstream of CATG site, and Illumina adaptor 2 was ligated at 3′end. Primer GX1 and primer GX2 were added for PCR. Then, 95 bp fragments through 6% TBE PAGE were regained. The cDNA was purified and followed by Illumina sequencing. Each tunnel would generate millions of raw reads with sequencing length of 35 bp. The DGE data of *P. splendens* RBB-5 at different stages of fermentation were submitted to gene expression omnibus (GEO) (accession number GSE43320), and the level of gene expression at the three stages of fermentation (48 h, 100 h and 148 h) were submitted to GEO respectively (corresponding accession number GSM1060479, GSM1060480 and GSM1060481).

### Analysis and mapping of DGE tags

The sequencing-received raw image data was transformed by base calling into sequence data. To map the DGE tags, the sequenced raw data was filtered to remove 3′adaptor sequence, empty tags (no tag sequence between the adaptors), low quality tags (tags with unknown nucleotide ‘N’), tags with only one copy number (which might result from sequencing errors) and tags (too long or too short). A virtual libraries containing all the possible CATG+17 bases length sequences of the reference gene sequences. All clean tags were mapped to the reference sequences and only 1 bp mismatch was considered. Clean tags mapped to reference sequences from multiple genes were filtered. For gene expression analysis, the number of unambiguous clean tags for each gene was calculated and then normalizes to TPM (number of transcripts per million clean tags) [39], [40].

### Evaluation of DGE libraries

We have developed a rigorous algorithm to identify differentially expressed genes among different fermentation stages using the method described by Audic et al [41]. False discovery rate (FDR) was a method to determine the threshold of P-Value in multiple test and analysis through manipulating the FDR value. We used ‘FDR≤0.001 and the absolute value of log_2_Ratio ≥1’ as the threshold to judge the significance of gene expression difference. For pathway enrichment analysis, we mapped all differentially expressed genes to terms in KEGG database and looked for significantly enriched KEGG terms compared to the genome background.

### Quantitative real-time PCR (qRT-PCR) validation

The sequences of the specific primer sets were listed in Additional File S1, and the 18S rRNA gene of *P. splendens*RBB-5 was used as an internal control. Quantitative Real-Time PCR (qRT-PCR) was performed using SYBR premix Ex Taq Kit (Takara, Japan) and ABI PRISM 7700 DNA sequence detection system (applied biosystems, USA) using the same cDNA samples as used with the RNA-seq experiment. A first-strand cDNA fragment was synthesized from total RNA treated with Dnase-I (Invitrogen) using superscript II reverse transcriptase (Invitrogen). The results were normalized to the expression level of the constitutive 18S rRNA gene. A relative quantitative method (ΔΔCt) was used to evaluate the quantitative variation. Each real-time PCR was carried out three times. The comparative threshold cycle method was used to calculate the relative gene expression [42].

### Lipid Extraction and fatty acid analysis by GC-MS

The total lipids extraction of cells and methyl esterification of fatty acids referred to the procedures described previously [20]. Fatty acid methyl esters (FAMEs) products were analyzed by GC-MS (Agilent). Fatty acids were identified by mass spectrum analysis.

## Results

### Illumina sequencing and reads assembly

**Figure 1:**
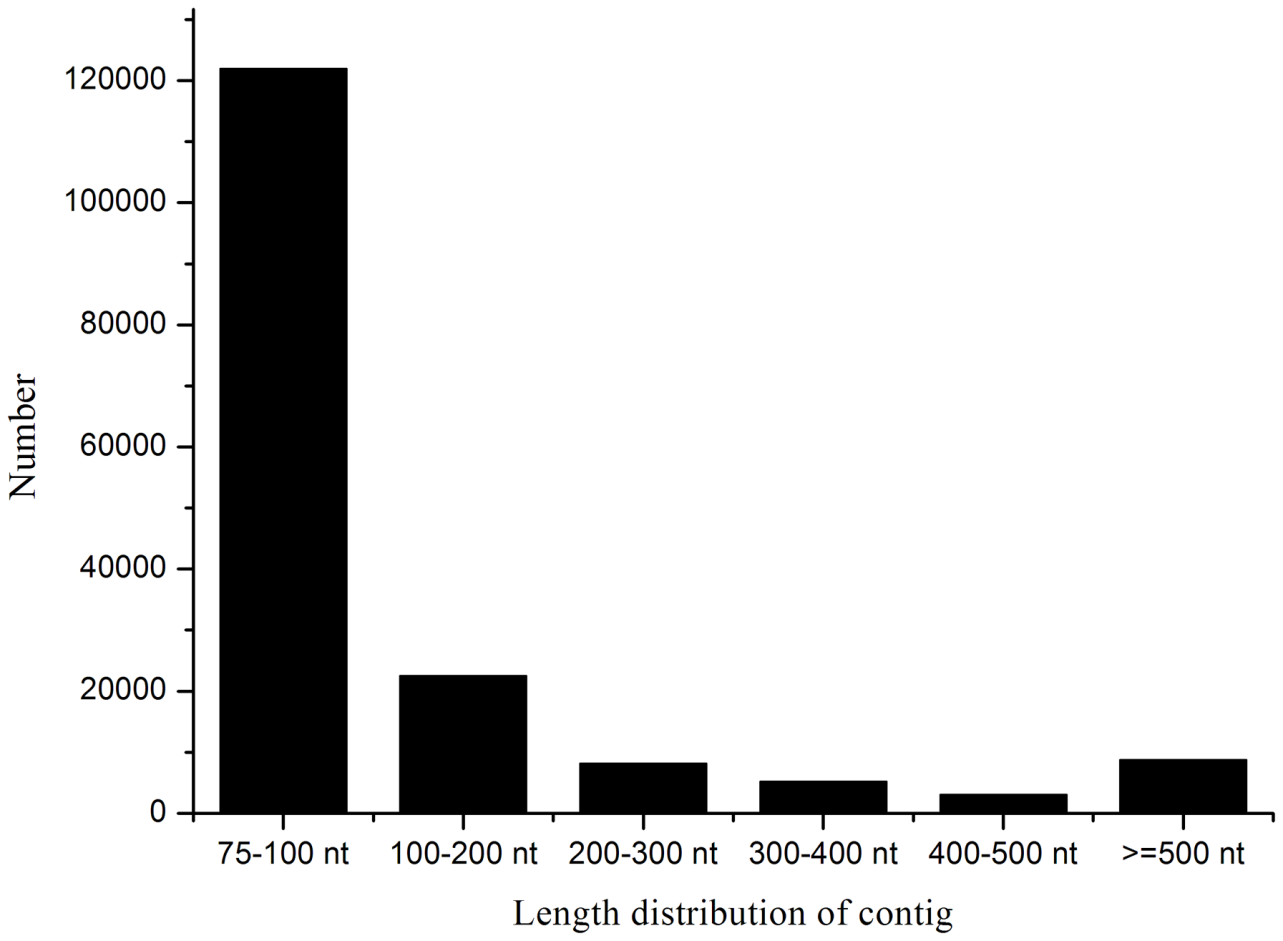
Contigs length distribution of *P. splendens*.

**Figure 2:**
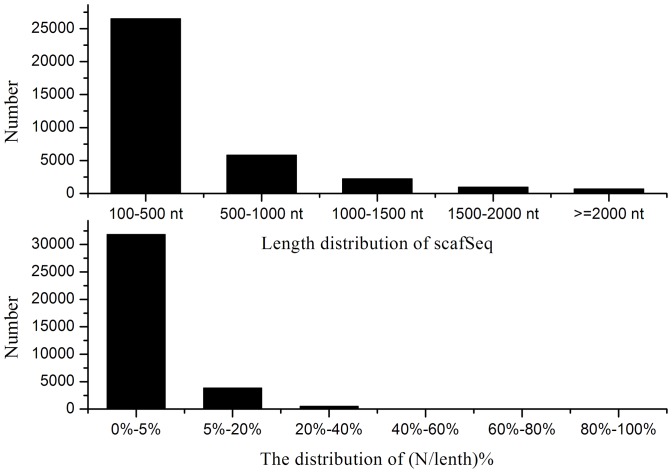
Scaffolds length distribution of *P. splendens*.

**Figure 3:**
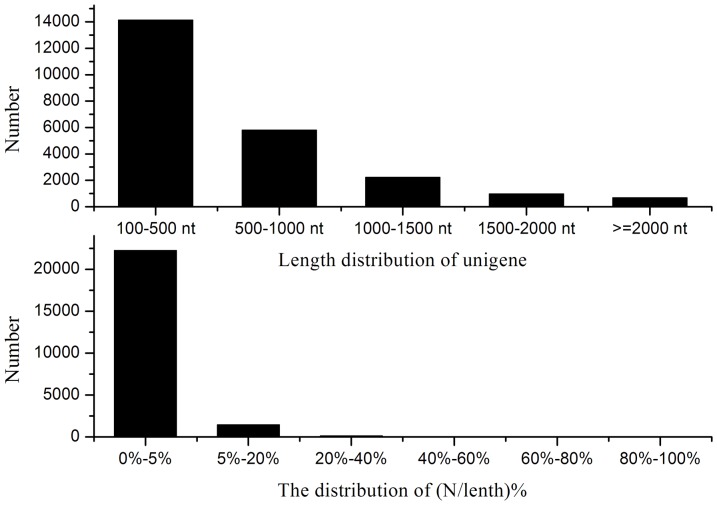
Unigenes length distribution of *P. splendens*

A cDNA sample was prepared at 48, 100 and 148 h and sequenced to obtain an overview of the *P. splendens*RBB-5 gene expression profile at different stages of fermentation. Illumina sequencing generated 13,288,892 reads (1,196,000,280 nt) after cleaning and quality checks; Q20 percentage (the percentage of bases with quality >20 in clean reads), N percentage and GC percentage are 82.54%, 0.00% and 56.16%, respectively. These reads were assembled randomly, producing 169,579 contigs with an N50 of 174 nt (i.e. 50% of the assembled bases were incorporated into contigs ≥174 nt) for *P. splendens*RBB-5 (Figure 1). Using paired end-joining and gap-filling, these contigs were further assembled using the SOAP denovo program [35] into 36,164 scaffolds with an N50 of 720 nt (3864 of which are >1000 nt) and a mean length of 447 nt (Figure 2). Finally, after gap filling using paired-end reads from the transcriptome data, TGICL software [43] was used to individually cluster these scaffolds, generating 23,796 unigenes. The assembly and contig joining succeeded in processing a large amount of short reads with relatively little redundancy. Figure 3 shows the unigene length distribution; 3866 unigenes have length >1000 nt (mean 610 nt) and an N50 of 828 nt (Figure 3).

We calculated the ratio of the gap length to the length of the assembled unigenes. The gap distribution (N/length%) <5% of the total length accounts for 93.47% of the unigenes. Three unigenes were selected and three pairs of primers for RT-PCR amplification were designed to demonstrate the quality of the sequencing data. All of the primer pairs produced bands of the expected sizes and the identity of the products is confirmed by sequencing.

### Annotation of predicted proteins

**Figure 4:**
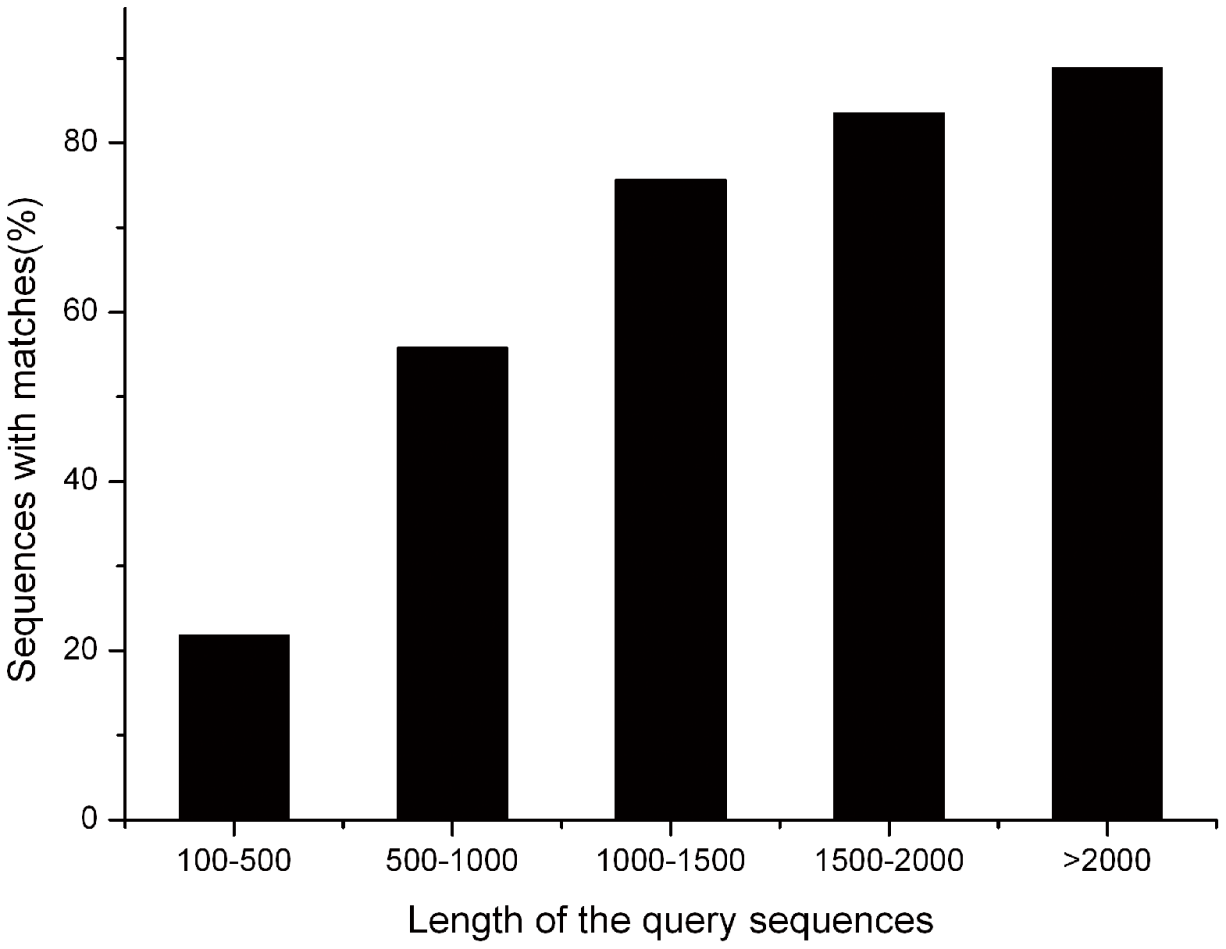
Effect of query sequence length on the percentage of sequences for which significant matches are found. The proportion of sequences with matches in NCBI nr databases is greater among the longer assembled sequences (with a cut-off E-value of 10^−5^).

**Figure 5:**
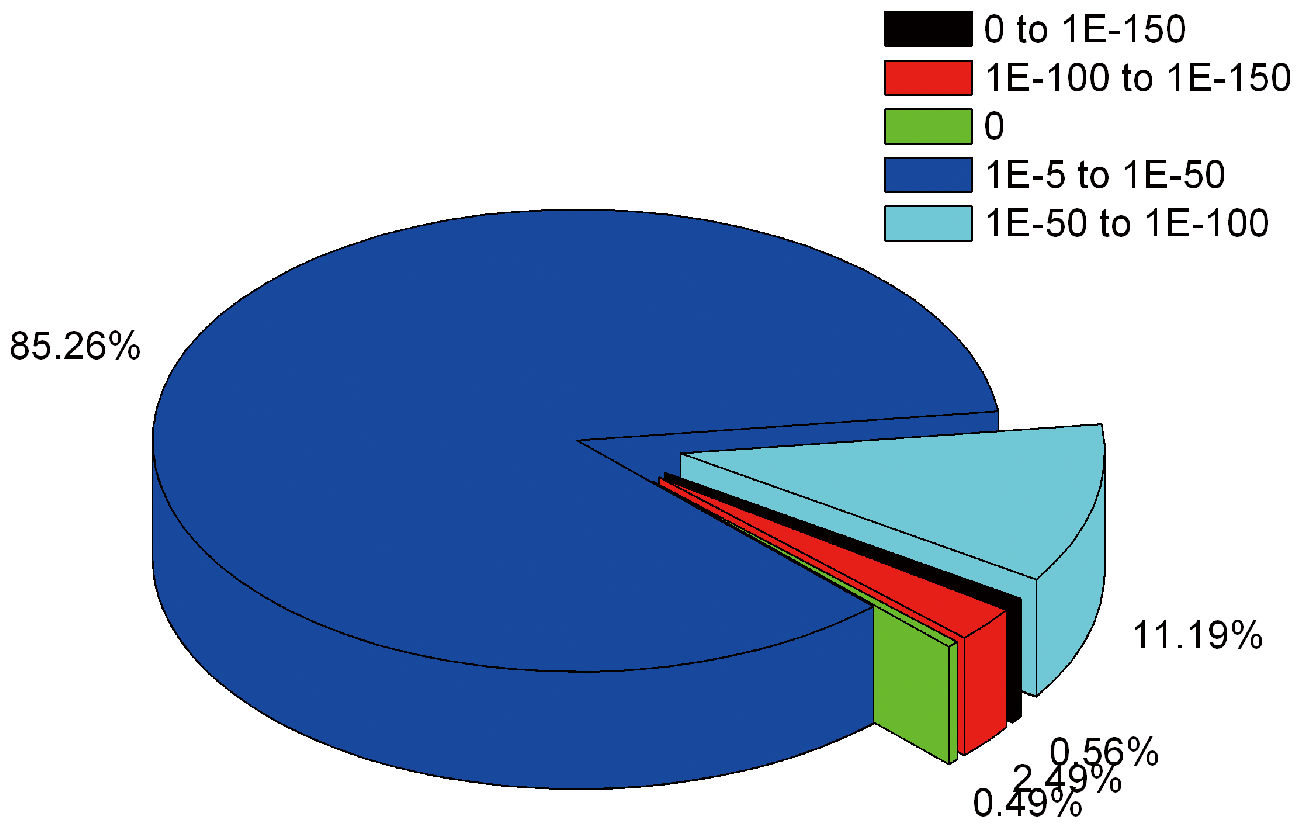
Characteristic of homology search of Illumina sequences against the nr database. E-value distribution of BLAST hits for each unique sequence with a cut-off E-value of 1.0 E^−5^.

For annotation, the *P. splendens*RBB-5 transcriptome sequences were searched using BLASTx against the non-redundant protein database (NR) in the NCBI nucleotide database with a cut-off *E*-value of 10^−5^. Using this approach, a total of 9398 unigenes (39.5% of all unigenes) matched known genes (Additional File S2). Owing to the lack of genome information for *P. splendens* and the relatively short length of distinct gene sequences (mean 610 nt), most of the 14,398 assembled sequences (60.5%) do not match known genes. The longer assembled sequences were better matched to known genes in the NR database (Figure 4). The match efficiency is 88.63% for sequences >2000 nt, 21.83% for sequences of 100–500 nt and 55.67% for sequences of 500–1000 nt (Figure 4). The *E*-value has strong homology (<1.0 E^−50^) in the NR database for ∼15% of the mapped sequences and 1.0 E^−5^ and 1.0 E^−50^ for the remainder (Figure 5).

### Classification of gene ontology (GO) and clusters of orthologous groups (COG)

**Figure 6:**
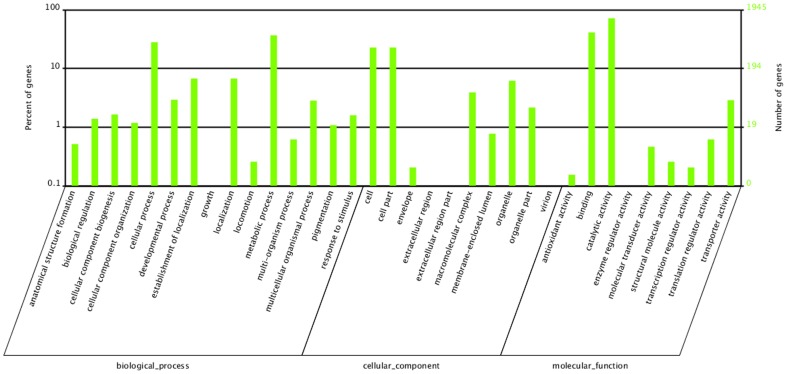
Classification of the gene ontology (GO) for the transcriptome of *P.splendens*. The results are summarized in three main categories: biological process, cellular component and molecular function. The right y-axis indicates the number of genes in a category. The left y-axis indicates the percentage of a specific category of genes in that main category.

**Figure 7:**
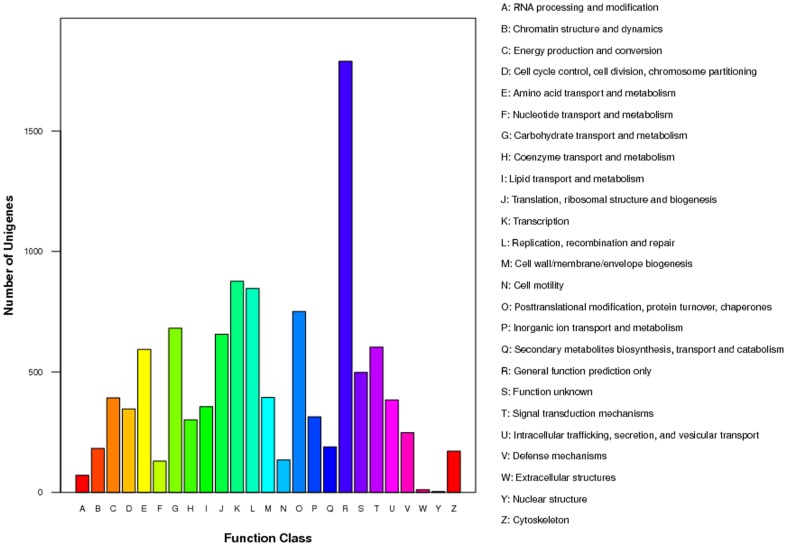
COG function classification of all unigenes from transcriptome of *P.splendens*. A, RNA processing and modification; B, Chromatin structure and dynamics; C, Energy production and conversion; D, Cell cycle control, cell division, chromosome partitioning; E, Amino acid transport and metabolism; F, Nucleotide transport and metabolism; G, Carbohydrate transport and metabolism; H, Coenzyme transport and metabolism; I, Lipid transport and metabolism; J, Translation, ribosomal structure and biogenesis; K, Transcription; L, Replication, recombination and repair; M, Cell wall/membrane/envelope biogenesis; N, Cell motility; O, Posttranslational modification, protein turnover, chaperones; P, Inorganic ion transport and metabolism; Q, Secondary metabolites biosynthesis, transport and catabolism; R, General function prediction only; S, Function unknown; T, Signal transduction mechanisms; U, Intracellular trafficking, secretion, and vesicular transport; V, Defense mechanisms; W, Extracellular structures; Y, Nuclear structure; Z, Cytoskeleton.

The GO terms were used to classify the function of the predicted *P.splendens* unigenes. On the basis of sequence homology, 9398 annotated unigenes were analyzed with Blast2GO for GO classification [36] and 5224 unigenes are categorized into 34 functional groups by WEGO [37] in the three ontologies molecular function, cellular component and biological process. The categories ‘metabolic processes, ‘cell part’, ‘cell’ and ‘catalytic activity’ are dominant. There is a high percentage of genes from categories ‘cellular process’ and ‘binding’ and only a few genes from categories ‘extracellular region’ and ‘virion’ (Figure 6). The annotated sequences for the genes involved in COG sprotein classifications were searched to further evaluate the completeness of the transcriptome library and the effectiveness of the annotation process. All of the 23,796 unigenes were aligned to the COGs database to predict and classify possible functions; 10,918 unigenes are annotated and divided into 25 specific categories, indicating a complicated transcriptome of *P. splendens* and providing a great deal of genetic information. Among the 25 specific categories, the cluster for ‘general function prediction only’ is the largest group (1789 (16.39%)) of unigenes, followed by ‘transcription’ (876, 8.02%), ‘replication, recombination and repair’ (846, 7.75%) and ‘post-translational modification, protein turnover, chaperones’ (751, 6.88%). The smallest group, ‘nuclear structure’, contains only three (0.027%) unigenes (Figure 7).

### Lipogenesis pathway

To identify the active biological pathways in *P. splendens*RBB-5, we mapped all unigenes in the kyoto encyclopedia of genes and genomes (KEGG) [44] database (the reference canonical pathways); 7300 unigenes are assigned to 158 KEGG pathways. Among them, the pathways most represented by the unique sequences were metabolic pathways (2178 members), biosynthesis of secondary metabolites (904 members) and urine metabolism (539 members). The lipid metabolic pathway of *P. splendens* was focused on because of its relatively low capacity to produce lipid. On the basis of the transcriptome information, lipogenesis pathways, including biosynthesis of fatty acids, unsaturated fatty acids, glycerolipids, glycerophospholipids, sterols etc. were studied (Additional File S3); 52 DEGs are annotated with biosynthesis of unsaturated fatty acids, 10 DEGs for fatty acid elongation in mitochondria and 192 DEGs for the glycolysis/gluconeogenesis pathway. The glycolysis pathway provides pyruvate, which is a key precursor for acetyl-CoA. There are 85 DEGs with annotation in the citrate cycle (TCA cycle) pathway and 11 unigenes are annotated with malic enzyme (ME). Some studies show over-expression of ME can increase fatty acid synthesis in fungi [45], suggesting ME is a major generator of NADPH; improving the NADPH level could increase lipid production in fungi. Among the enzymes involved in fatty acid synthesis directly (52 members), acetyl-CoA carboxylase (7 members) converts acetyl-CoA to malonyl-CoA, which is a rate-limiting step in fatty acid biosynthesis, and fatty acid synthase (13 members), is necessary for the synthesis of saturated fatty acids (e.g. octanoic acid, dodecanoic acid and hexadecanoic acid) from acetyl-CoA and malonyl-CoA (Additional File S4). Malonyl-CoA decarboxylase, which counters acetyl-CoA carboxylase action, was not found in fatty acid biosynthesis. If there is no enzyme with malonyl-CoA decarboxylase function in *P. splendens*, this could increase the levels of malonyl-CoA and favor lipid synthesis.

### DGE library sequencing

**Figure 8:**
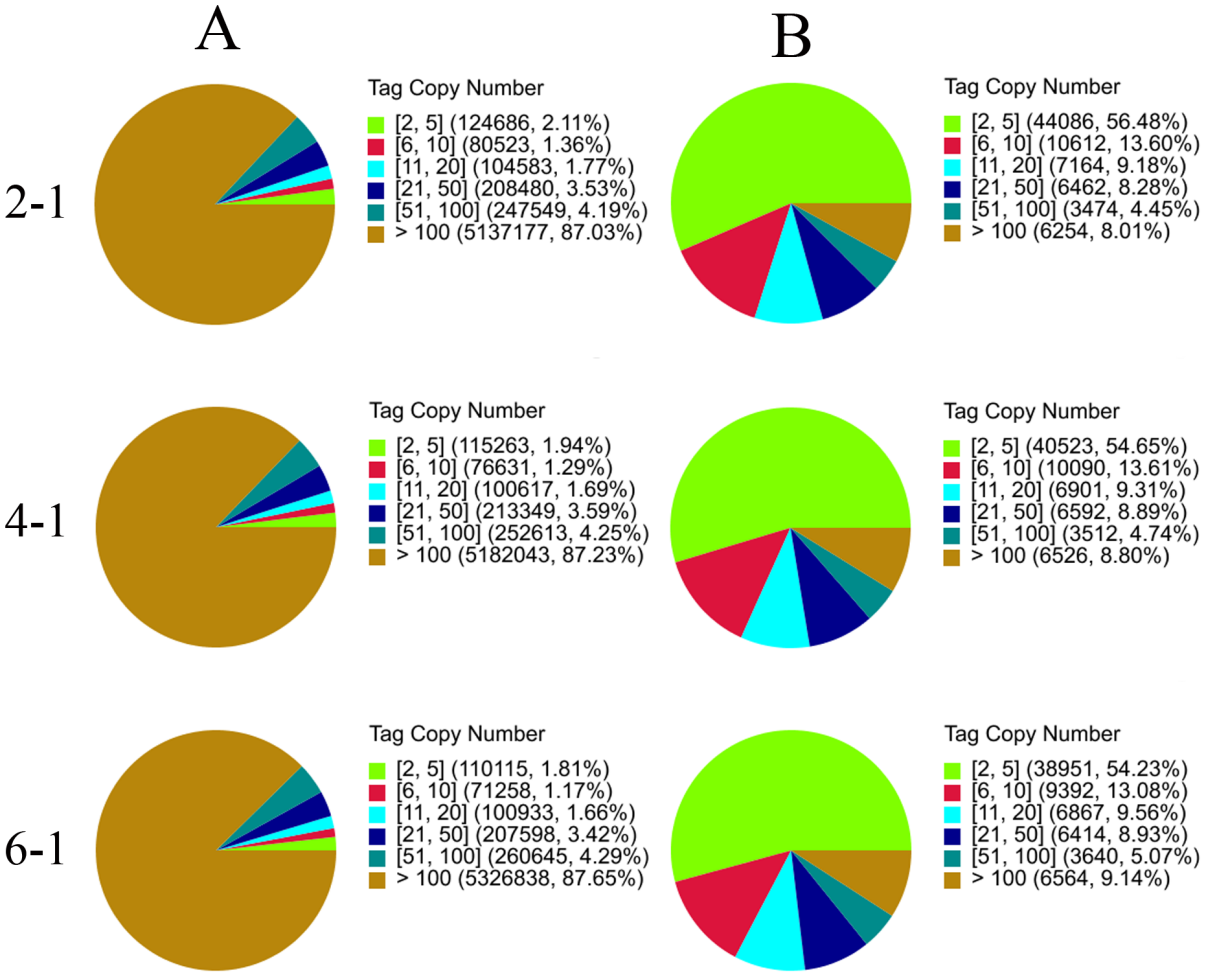
Distribution of total clean tags and distinct clean tags in each sample (2-1: 48 h, 4-1: 100 h, 6-1: 148 h). (A) Distribution of total clean tags (a: sample 2-1; b: sample 4-1; c: sample 6-1). (B) Distribution of distinct clean tags (a: sample 2-1; b: sample 4-1; c: sample 6-1).

An immediate application of the transcriptome sequence data is in gene expression profiling characterization at different stages of fermentation. The DGE method generates an absolute measurement rather than relative gene expression and avoids many of the inherent limitations of microarray analysis. Variations of *P.splendens* gene expression were analyzed during different stages of fermentation. Three *P.splendens* DGE libraries (48, 100 and 144 h of fermentation) are sequenced, generating ∼6×10^6^ raw tags for each of the three libraries. When the low-quality tags are removed, the number of clean tags per library ranges from 5.9×10^6^–6.08×10^6^ (Table 1) and the percentage of clean tags in each library among the raw tags ranges from 98.16–98.46%. The number of sequences mapped to unigenes ranges from 1.6×10^6^–1.8×10^6^ among the clean tags (Table 1). Heterogeneity and redundancy are important characteristics for mRNA expression. A small number of mRNA categories have a high level of expression but most stay at a very low level. So, the normality of the DGE data is evaluated via the distribution of clean tag expression. The high-expression tags with copy numbers >100 showed values >85%, whereas the distribution of distinct clean tags did not exceed 10% in each library (Figure 8). By contrast, the low-expression genes with copy numbers <5 have a broad distribution of distinct clean tags in each library.

**Table 1:** Tag analysis statistics.

Summary		48 h	100 h	148 h
Raw Data	Total number	6013524	6033685	6172287
	Distinct number	186452	167215	166604
Clean Tag	Total number	5902998	5940516	6077387
	Distinct number	78052	74144	71828
All Tag Mapping to Gene	Total number	1738545	1822236	1639848
	Total of Clean Tag (%)	29.45%	30.67%	26.98%
	Distinct number	33362	30539	27819
	Total of Clean Tag	42.74%	41.19%	38.73%
Unambiguous Tag Mapping to Gene	Total number (%)	1737986	1821693	1639377
	Total of Clean Tag (%)	29.44%	30.67%	26.98%
	Distinct number	33324	30495	27786
	Distinct of Clean Tag (%)	42.69%	41.13%	38.68%
All Tag-mapped Genes	Total number	12540	12112	11516
	Total of ref genes (%)	52.70%	50.90%	48.39%
Unambiguous Tag-mapped Genes	Total number	12523	12092	11495
	Total of ref genes (%)	52.63%	50.82%	48.31%
Unknown Tag	Total number	4164453	4118280	4437539
	Total of Clean Tag (%)	70.55%	69.33%	73.02%
	Distinct number	44690	43605	44009
	Distinct of Clean Tag (%)	57.26%	58.81%	61.27%

### Mapping sequences to the reference transcriptome database

**Figure 9:**
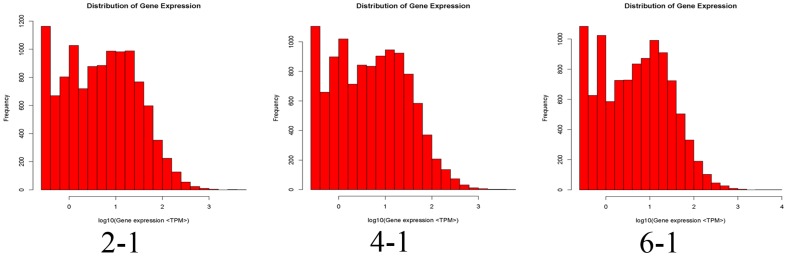
The level of gene expression for each gene (a: sample 2-1, b: sample 4-1, c: sample 6-1). Gene expression level was determined by calculating the number of unambiguous tags for each gene and then normalizing to TPM (transcript copies per million tags).

According to our transcriptome reference database generated by Illumina sequencing, we mapped the tag sequences of the three DGE libraries to reveal the molecular events behind the DGE profiles. The reference database contains 67,398 tags with 67,282 unambiguous reference tags. Among the 71,828–78,052 distinct tags generated from the Illumina sequencing of the three DGE libraries, 27,786–73,555 distinct tags were mapped to genes in the reference database (Table 1). The sequences up to 12,523 (52.63%) in the database of our transcriptome reference tag are identified explicitly by unique tag (Table 1). A saturation analysis checked whether the number of detected genes continues to increase when sequencing amount (total tag number) increases. The number of unambiguous tags was calculated for each gene to determine the level of gene expression and normalized to the number of transcript per million clean tags (TPM), which shows more genes are represented in <10 copies, and relatively less in the highly expressed genes (Figure 9).

### Comparison of gene expression profile among different fermentation stages

The number of clean tags for each gene between the two samples was calculated using an algorithm developed by Audic *et al.* [41] to identify the genes expressed differentially among different stages of fermentation. We list the top 20 most abundantly expressed genes with annotation from the three libraries (Additional File S5). We noticed the presence of annex in ANXC3.2 in all three libraries and the expression was highest in the 48 h libraries. The annexins expressed widely in eukaryotes are Ca^2+^ and phospholipid-binding proteins, which form an evolutionarily conserved multigene family, and the family proteins are implicated in a wide range of important biological processes, including membrane–cytoskeleton interaction, cell growth control, ion channel regulation and signal transduction, so they are important in cell growth. We found that glycerol-3-phosphate dehydrogenase (GPDH) is expressed abundantly only in the 100 h library andglyceraldehyde-3-phosphate dehydrogenase (GAPDH) is expressed abundantly in the 100 h and 148 h libraries, which suggests growth and lipid biosynthesis are related to GAPDH and GPDH directly. Some studies have revealed that GAPDH, which converts glycerol dehyde 3-phosphate to 1,3-bisphosphoglycerate has a crucial role in glucose catabolism. The most common form is the NAD^+^-dependent enzyme found in all organisms and located usually in the cytoplasm [46]. GPDH has an important role in energy metabolism and regulates the ratio of cytoplasmic NAD^+^/NADH [47]. A larger amount of NADH could increase final cell density and provoke a significant change in the final pattern of metabolite concentration, and the availability of NADH could be increased by adding formate to the medium [48]. GPDH activity is regulated through lipid–enzyme interactions [49] and it is the rate-limiting enzyme for glycerol synthesis [50], [51]. Thus, regulation of GAPDH and GPDH gene expression should improve the yields of EPA.

**Figure 10:**
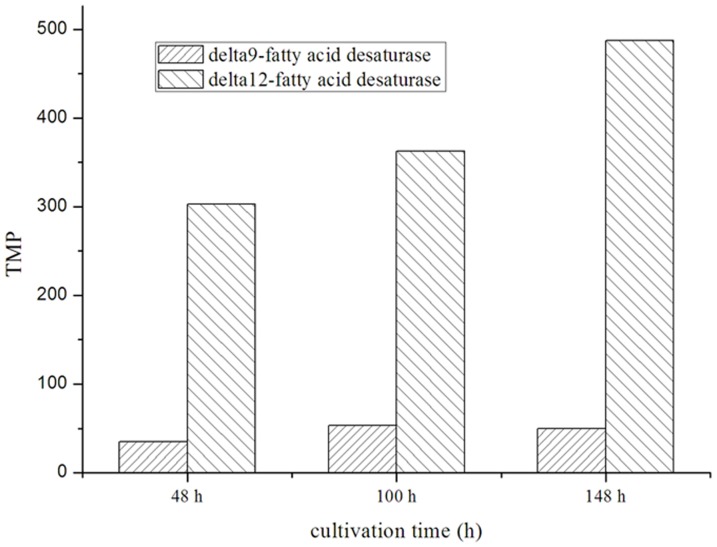
The gene expression of D12 and D9 based on the data of DGE among different fermentation stages of *P. splendens*.

**Figure 11:**
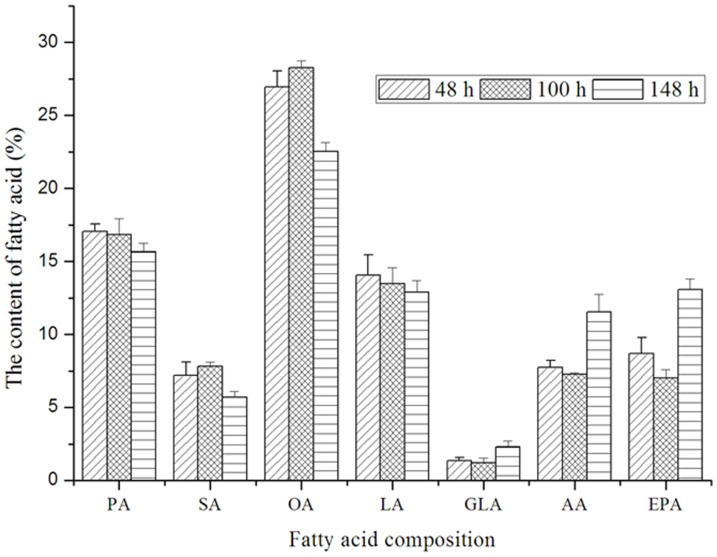
The fatty acid composition of *P. splendens* at different fermentation stages. PA: palmitic acid, SA: stearic acid, OA: oleic acid, LA: linoleic acid, GLA: γ-linolenic acid, AA: arachidonic acid, EPA: eicosapentaenoic acid.

We examined expression of the gene for Δ12 fatty acid desaturase (D12) and Δ9 fatty acid desaturase (D9) and the relationship between gene expression and fatty acid biosynthesis. On the basis of the data for DGE, expression of the genes for D12 and D9 are shown in Figure 10 and the fatty acid composition is shown in Figure 11. According to the fatty acid composition, the stearic acid and oleic acid content decrease with cultivation time, whereas expression of the genes for D9 and D12 increases (Figure 10), indicating change of fatty acid composition is related to gene expression. The level of expression for the D12 gene was high, but accumulation of oleic acid was greater, so we studied the enzyme activity of D12 further. The content of oleic acid was decreased further by adding the divalent metal ions Zn^2+^ and Mn^2+^ (data not shown). These results show fatty acid composition is regulated by gene expression and enzyme activity.

Cytochrome P450 enzymes (P450s) are heme–thiolate proteins involved in the oxidative degradation of a variety of compounds with crucial roles in fungal biology and ecology. More than 6000 fungal genes coding for putative P450s from 276 families are identified [52] and 50 *Pythium* cytochrome P450 genes was identified in this study. Protein BLAST analysis of *Pythium* P450s showed homology between 78A3, 86A2, 86B1, 90A, 94A1 and 704C1 P450 family members and other cytochrome P450-related domains. We analyzed the CYPs further, the NADPH-P450 reductase was a key component in the respiratory chain, and its function is similar to the cytochrome P450 family 505, which has a strong preference for NADPH over NADH as the electron donor, function, catalytic rate in other organisms (*Fusarium oxysporum*) [53], so it may be related to EPA production. And the level of NADPH-P450 reductase gene expression was the lowest in the libraries of 100 h than 48 h and 148 h.

**Figure 12:**
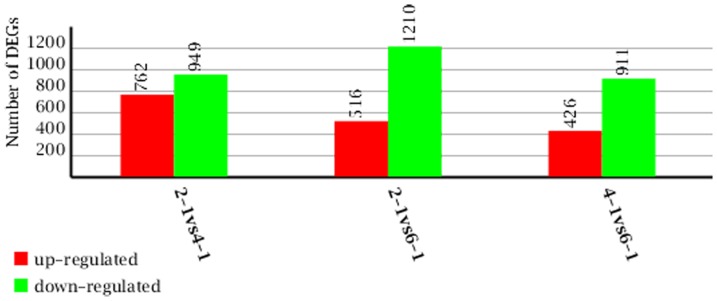
Numbers of DGE unigenes in each comparison (2-1: 48 h, 4-1: 100 h, 6-1: 148 h). Up-(red) and down-regulated (green) unigenes were quantified.

The three stages of fermentation were further evaluated in three pair wise comparisons: 48 h vs. 100 h, 48 h vs. 148 h and 100 h vs. 148 h. Of 1726 genes, 516 were up-regulated and 1210 were down-regulated, with a significant difference of expression levels at 48 h and 148 h of fermentation (Figure 12). Giving insight into the 20 biggest differences of gene expression with annotation between 48 h and 148 h of fermentation (Additional File S6), there is a specific enrichment of genes for pathways involved in energy and metabolism at 148 h [54], [55], such as short-chain dehydrogenase, triacylglycerol lipase and phosphoenol pyruvatecarb oxykinase (GTP). GTP is a key enzyme in the synthesis of glyceride/glycerol in particular [56], but the level of gene expression is relatively low, likely indicating the rate-limiting enzyme of *P. splendens* lipid biosynthesis is GTP. Out of a total of 1711 genes, 762 are up-regulated and 949 are down-regulated at 100 h compared to 48 h (Figure 12). The top ten largest differences of gene expression with annotation between 48 h and 100 h were studied further (Additional File S7). There is a specific increase of enzyme activity associated with energy metabolism at 48 h [57], including phospho lipid-transporting ATPase and phosphate acetyl transferase as well as fatty acid synthase alpha subunit [58], which has an important role in de novo synthesis of fatty acids. Fatty acids are key components of the cell and their synthesis is essential for all organisms. Out of a total of 1337 genes, 426 are up-regulated and 911 genes are down-regulated at 148 h (Figure 12). The top ten largest differences of gene expression with annotation between 100 h and 148 h were studied further (Additional File S8), at 100 h, the genes were focused on stress reaction, such as alanine amino transferase and Chd1p.

Further cluster analysis of the union of differentially expressed genes (48 h vs. 100 h, 100 h vs. 148 h and 48 h vs. 148 h) was under taken. Study of genes expressed differentially in mitochondrial fatty acid elongation, fatty acid biosynthesis and biosynthesis of unsaturated fatty acids showed genes related to lipid biosynthesis and fatty acid biosynthesis (fatty acid synthase subunit alpha, fatty acid synthase alpha-subunit reductase and acyl-CoA oxidase) are not in the same gene cluster, but they have similar levels of expression and could be co-regulated.

### Functional gene expression profile among different stages of fermentation

The three stages of fermentation were further evaluated on the basis of GO classification; genes with annotation with the highest level of expression at 100 h of fermentation are associated with the fatty acid metabolic process and the glycerol 3-phosphate metabolic process, which are concentrated in lipid biosynthesis (Additional File S9). The fatty acid metabolic process is observed at the three stages of fermentation; the level of gene expression is highest at 148 h, indicating that the rate of fatty acid conversion is faster at 148 h than it is at 48 h and 100 h (Additional File S9). Further comparison of gene expression at the three stages showed a large number of DEGs with annotation were dominant in categories ‘cell’, ‘metabolic process’, ‘catalytic activity’ and ‘cellular metabolic process’ (Additional Files S10, S11 and S12). Then, on the basis of the whole transcriptome sequence, all DEGs were mapped in the KEGG database to search for genes involved in metabolic or signal transduction pathways (Additional File S13). Among all the genes with KEGG pathways annotation, 552 are identified with genes expressed differently at 48 h and 148 h of fermentation. Specific enrichment of genes is observed for pathways involved in metabolic pathways (205 DGEs, 37.14%) and biosynthesis of secondary metabolites (101 DGEs, 18.3%), such as butanoate metabolism, benzoate degradation via hydroxylation and 1- and 2-methylnaphthalene degradation. The secondary metabolites are increased at 148 h, likely indicating metabolic flux is changed, leading to the waste of energy. A total of 590 genes expressed differently at 48 h and 100 h are identified and a specific enrichment of energy and primary metabolite biosynthesis is observed; e.g. pantothenate and CoA biosynthesis, which is an important role in energy metabolism, valine, leucine and isoleucine and aminoacyl-tRNA biosynthesis, which have an important role in the growth of *P.splendens*. The biosynthesis of steroids in cell membranes is increased, which might be a response to maintain the fluidity of the membrane at low temperature (15°C). A total of 420 differently expressed genes are identified and there is a specific enrichment of the biosynthesis of secondary metabolites between 100 h and 148 h. There is also an enrichment of differently expressed genes related to lipid metabolism, such as fatty acid metabolism and glycerolipid metabolism, which likely indicates the level of lipid metabolism in *P.splendens*RBB-5 at 100 h or 148 h is higher than that at 48 h. Glycolysis/gluconeogenesis was also found at the libraries of 100 h and 148 h, likely indicating dissolved oxygen is deficient at 148 h according to the high density of *P.splendens*. This annotation of genes at different stages of fermentation provides a valuable resource for investigating specific processes, functions and pathways in *P.splendens*.

### Validation of gene expression profile by qRT-PCR

**Figure 13:**
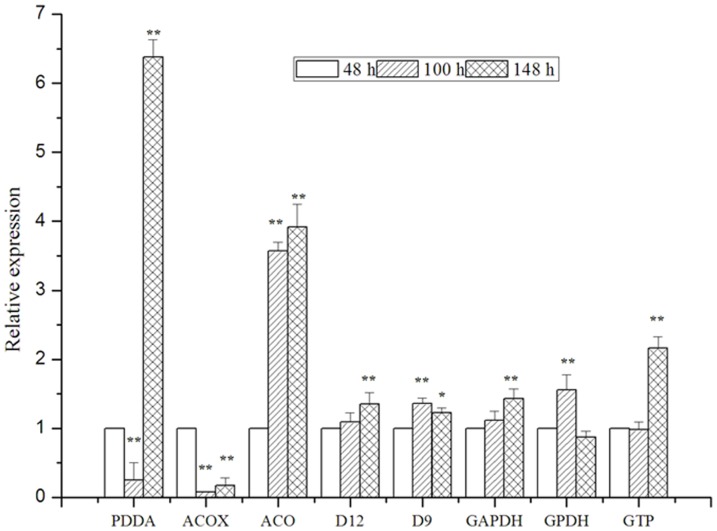
Validation of the gene expression profile by qRT-PCR. PDDA: phospho-2-dehydro-3-deoxyheptonate aldolase, ACOX: acyl-coenzyme an oxidase, ACO: aconitate hydratase, D12: Δ12 fatty acid desaturase, D9: Δ9 fatty acid desaturase, GAPDH: Glyceraldehyde-3-phosphate dehydrogenase, GPDH: Glycerol-3-phosphate dehydrogenase, GTP: phosphoenolpyruvate carboxykinase; *P<0.05, **P<0.01.

Eight genes (phospho-2-dehydro-3-deoxyheptonatealdolase, acyl-CoA, aconitatehydratase, D12, D9, GAPDH, GPDH and GTP) associated with lipid and fatty acid biosynthesis at 48, 100 and 148 h were selected to evaluate the DGE data further and to confirm the gene expression profiles. The qRT-PCR expression profiles of the eight genes are consistent with the DGE results (Figure 13), further supporting the reliability of the RNA-Seq data.

## Discussion

Microbial oil is a good source of dietary fat and energy and EPA is an essential PUFA. *Pythium* is potentially suitable for high yield EPA production. Many researchers focused on the study of EPA production using microorganisms such as *P. irregular* [59], [60] and we have done some research with *P. splendens*RBB-5. We found the temperature was important for EPA production; the yield of EPA is greatest at 25°C in the first four days and at 15°C in the last three days and these results are also confirmed by Yi Liang, et al. [60], but the metabolic pathway and key genes at the different stages of fermentation in *P. splendens*RBB-5 remained unknown. Therefore, elucidation of the mechanisms underlying cell growth and EPA yield and identification of new genes responsible for EPA biosynthesis is important. This study shows *P. splendens*RBB-5 accumulates a large amount of linoleic acid (C18:2) as triacylglycerols in the mycelia, and the oleic acid (C18:1) and linoleic acid (C18:2) are >75% of total fatty acids. However, few studies have revealed details of the mechanisms responsible for the synthesis of lipid and few genes encoding key enzymes of EPA synthesis are identified. So, there is an urgent need to find the key step in lipid synthesis. Three different stages of growth (48, 100 and 148 h of fermentation) were chosen to study gene expression using the transcriptome sequence and DGE analysis.

In this study, transcriptome and DGE analysis using Illumina sequencing technology produced more than 23,792 genes with 9398 having an above cut-off BLAST result. One objective was to find an efficient way to control the metabolic processes of *P.splendens*. We analyzed our transcriptome database and identified the most important enzymes associated with the metabolism of lipids or genes regulating the biosynthesis of EPA. Triacylglycerol (TAG) is the major lipid class in oleaginous microorganisms, such as AA and EPA [61], [62]. On the basis of the GO classification, fatty acid metabolic processes are enriched significantly at the three chosen stages; the glycerol 3-phosphate metabolic process is particularly enriched at 100 h. The results showed a relationship between the enriched pathways and fermentation stages, consistent with the expectation of the enrichment of pathways at different stages of fermentation in *P. splendens*.

These results are a substantial contribution to finding new sequences of *P. splendens* and most known key genes involved in lipid biosynthesis were found, including Δ6, Δ17 and Δ9 fatty acid desaturase genes, fatty acid elongase and fatty acid synthase. The entire sequences of the Δ6 and Δ17 fatty acid desaturase genes were obtained from the transcriptome data and these will be published elsewhere. It should be pointed out that although a large number of potentially interesting genes were obtained from the transcriptome data, most of them were partial sequences of specific genes, and some sequences were excluded from the analysis because they are very short or have poor alignment. Currently, RACE technology is the preferred choice for obtaining the full sequence of these genes.

To our knowledge, this is the first report of the transcriptome of *P. splendens* without prior genome annotation. Transcriptome and DGE analysis using Illumina sequencing technology initially revealed differential expression of genes and metabolic pathways at different stages of fermentation, and some key genes encoding enzymes associated with lipid biosynthesis are identified. The genome of *Phytophthora*, which is in the same family as *Pythium*, has been reported [63]; although the aims of that study are different, it might provide some basic genetic information for further studies of *P.splendens*.

## Conclusions

Using Illumina sequencing technology, we analyzed the *P. splendens* transcriptome and which produced 23,796 assembled unigenes. By comparison to known genes from other microbial species, 9398 unigenes are annotated. The *P. splendens* transcriptome and DGE profiling data greatly expand the amount of genetic information available for *Pythium* and provide a profile of different stages of fermentation. In summary, the transcriptome data serve as an important public information platform to accelerate research into metabolic networks of *P. splendens* and the Illumina sequencing-based DGE system for gene expression profiling reveals the relationship between the enriched pathways and fermentation stages. This study is a first step toward a better understanding of the functions of these genes and provides a broad new vision of the future of study at the molecular level.

## Supporting Information

Additional File S1 **Primers used in qRT-PCR for validation of differentially expressed genes.**
(XLSX)

Additional File S2 **The annotation of all unigenes.**
(XLSX)

Additional File S3 **Unigenes with pathway annotation.**
(XLSX)

Additional File S4 **DGEs with annotation in fatty acid biosynthesis.**
(XLSX)

Additional File S5 **The top 20 most abundantly expressed genes with annotation from the three libraries 48 h, 100 h and 148 h.**
(XLSX)

Additional File S6 **Differentially expressed genes between 48 h and 148 h (2-1 and 6-1).**
(XLSX)

Additional File S7 **Differentially expressed genes between 48 h and 100 h (2-1 and 4-1).**
(XLSX)

Additional File S8 **Differentially expressed genes between 100 h and 148 h (4-1 and 6-1).**
(XLSX)

Additional File S9 **Analyses of GO process based on the high level of gene expression in the three libraries 48 h, 100 h and 148 h.**
(XLSX)

Additional File S10 **Comparison of GO classification between 48 h and 100 h.**
(XLSX)

Additional File S11 **Comparison of GO classification between 48 h and 148 h.**
(XLSX)

Additional File S12 **Comparison of GO classification between 100 h and 148 h.**
(XLSX)

Additional File S13 **Analyses of pathways in the three comparsion libraries (48 h vs. 100 h, 48 h vs. 148 h and 100 h vs. 148 h).**
(XLSX)
